# Benzamidinium 2-meth­oxy­benzoate

**DOI:** 10.1107/S1600536813016395

**Published:** 2013-06-19

**Authors:** Gustavo Portalone

**Affiliations:** a Chemistry Department, "Sapienza" University of Rome, P.le A. Moro, 5, I-00185 Rome, Italy

## Abstract

The title mol­ecular salt, C_7_H_9_N_2_^+.^C_8_H_7_O_3_^−^, was synthesized by reaction between benzamidine (benzene­carboximidamide) and 2-meth­oxy­benzoic acid. In the cation, the amidinium group has two similar C—N bonds [1.3070 (17) and 1.3145 (16) Å] and is almost coplanar with the benzene ring, making a dihedral angle of 5.34 (12)°. In the anion, the meth­oxy substituent forces the carboxyl­ate group to be twisted by 69.45 (6)° with respect to the plane of the aromatic fragment. In the crystal, the components are connected by two N^+^—H⋯O^−^ (±)CAHB (charge-assisted hydrogen bonds), forming centrosymmetric ionic dimers with graph-set motif *R*_2_^2^(8). These ionic dimers are then joined in ribbons running along the *b-*axis direction by another *R*_4_^2^(8) motif involving the remaining N^+^—H⋯O^−^ hydrogen bonds. Remarkably, at variance with the well known carb­oxy­lic dimer *R*_2_^2^(8) motif, the carboxyl­ate–amidinium pair is not planar, the dihedral angle between the planes defined by the CN_2_^+^ and CO_2_^−^ atoms being 18.57 (12)°.

## Related literature

For the biological and pharmacological relevance of benzamidine, see: Powers & Harper (1999 ▶). For structural analysis of proton-transfer adducts containing mol­ecules of biological inter­est, see: Portalone (2010 ▶, 2013 ▶). For the supra­molecular association in proton-transfer adducts containing benzamidinium cations, see: Portalone (2010 ▶, 2012 ▶, 2013 ▶); Irrera & Portalone (2012 ▶, 2013 ▶); Irrera *et al.* (2012 ▶). For 2-meth­oxy­benzoic acid derivatives, see: Portalone (2011 ▶). For hydrogen-bond motifs, see: Bernstein *et al.* (1995 ▶).
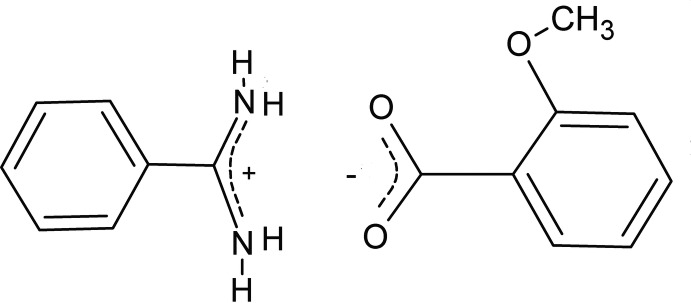


## Experimental

### 

#### Crystal data

C_7_H_9_N_2_^+^·C_8_H_7_O_3_^−^
*M* *_r_* = 272.30
Triclinic, 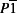

*a* = 7.5154 (3) Å
*b* = 9.1393 (4) Å
*c* = 11.6498 (5) Å
α = 69.612 (3)°
β = 80.500 (5)°
γ = 72.482 (4)°
*V* = 713.63 (6) Å^3^
*Z* = 2
Mo *K*α radiation
μ = 0.09 mm^−1^
*T* = 298 K
0.12 × 0.09 × 0.05 mm

#### Data collection

Agilent Xcalibur Sapphire3 diffractometer
Absorption correction: multi-scan (*CrysAlis PRO*; Agilent, 2011 ▶) *T*_min_ = 0.989, *T*_max_ = 0.996
20559 measured reflections
4332 independent reflections
3188 reflections with *I* > 2σ(*I*)
*R*_int_ = 0.025

#### Refinement

*R*[*F* ^2^ > 2σ(*F* ^2^)] = 0.062
*wR*(*F* ^2^) = 0.161
*S* = 1.05
4332 reflections
199 parameters
H atoms treated by a mixture of independent and constrained refinement
Δρ_max_ = 0.38 e Å^−3^
Δρ_min_ = −0.18 e Å^−3^

### 

Data collection: *CrysAlis PRO* (Agilent, 2011 ▶); cell refinement: *CrysAlis PRO*; data reduction: *CrysAlis PRO*; program(s) used to solve structure: *SIR97* (Altomare *et al.*, 1999 ▶); program(s) used to refine structure: *SHELXL97* (Sheldrick, 2008 ▶); molecular graphics: *ORTEP-3 for Windows* (Farrugia, 2012 ▶); software used to prepare material for publication: *WinGX* (Farrugia, 2012 ▶).

## Supplementary Material

Crystal structure: contains datablock(s) global, I. DOI: 10.1107/S1600536813016395/nk2209sup1.cif

Structure factors: contains datablock(s) I. DOI: 10.1107/S1600536813016395/nk2209Isup2.hkl

Additional supplementary materials:  crystallographic information; 3D view; checkCIF report

## Figures and Tables

**Table 1:** Hydrogen-bond geometry (Å, °)

*D*—H⋯*A*	*D*—H	H⋯*A*	*D*⋯*A*	*D*—H⋯*A*
N1—H1*A*⋯O1	0.913 (19)	1.87 (2)	2.7777 (16)	172.4 (17)
N1—H1*B*⋯O1^i^	0.87 (2)	1.97 (2)	2.7926 (15)	155.7 (17)
N2—H2*A*⋯O2	0.959 (19)	1.93 (2)	2.8863 (17)	175.4 (16)
N2—H2*B*⋯O2^ii^	0.86 (2)	2.00 (2)	2.8230 (16)	160.3 (18)

Symmetry codes: (i) 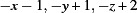; (ii) 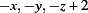.

**Fig. 1.**
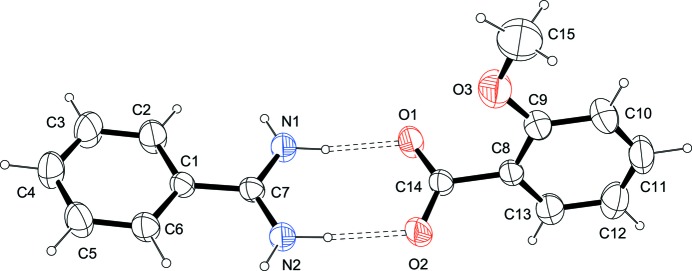
The asymmetric unit of (I), showing the atom-labelling scheme. Displacements ellipsoids are at the 50% probability level. The asymmetric unit was selected so that the two ions are linked by N—H+···O- hydrogen bonds. H atoms are shown as small spheres of arbitrary radii.

**Fig. 2.**
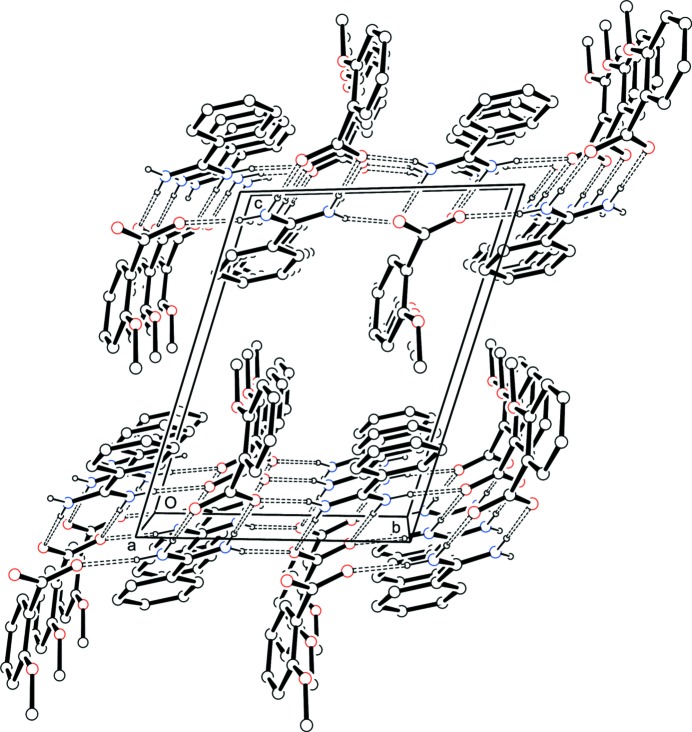
Crystal packing diagram for (I), viewed approximately down a. All atoms are shown as small spheres of arbitrary radii. For the sake of clarity, H atoms not involved in hydrogen bonding have been omitted. Hydrogen bonding is indicated by dashed lines.

## supplementary crystallographic information

### Comment

The present study is a continuation of the work carried out in this Laboratory on proton-transfer adducts containing molecules of biological interest (Portalone, 2010, 2013), and deals with the single-crystal structure of the molecular salt, benzamidinium 2-methoxybenzoate, (I), obtained by a reaction between benzamidine (benzenecarboximidamide) and 2-methoxybenzoic acid in water solution. Benzamidine derivatives, which have shown strong biological and pharmacological activity (Powers & Harper, 1999), are being used in this Laboratory as bricks for supramolecular construction (Portalone, 2010). Indeed, these molecules are strong Lewis base and their cations can be easily anchored onto numerous inorganic and organic anions and polyanions, largely because of the presence of four potential donor sites for hydrogen-bonding.

The asymmetric unit of (I) comprises one planar benzamidinium cation and one 2-methoxybenzoate anion (Fig. 1).

In the cation the amidinium group forms a dihedral angle of 5.34 (12)° with the mean plane of the phenyl ring, at variance with the values observed in protonated benzamidinium ions (23.2 - 31.1°, Portalone, 2010; Portalone, 2013; Irrera *et al.*, 2012; Irrera & Portalone, 2012, 2013). The pattern of bond lengths and bond angles of the benzamidinium cation agrees with that reported in previous structural investigations. In particular the amidinium group, true to one's expectations, features similar C—N bonds [1.3070 (17) and 1.3145 (16) Å], evidencing the delocalization of the π electrons and double-bond character.

In the 2-methoxybenzoate anion the benzene ring is essentially planar and the methoxy substituent forces the carboxylate group to be twisted with respect to the plane of the aromatic fragment by 69.45 (6)°. In the anion bond lengths and bond angles of the benzene ring are in accord with corresponding values obtained for both the orthorhombic and tetragonal forms of 2,6-dimethoxybenzoic acid (Portalone, 2011 and reference therein) and 4-methoxybenzamidinium 2,6-dimethoxybenzoate (Portalone, 2012). The C—O distances of the carboxylate group, 1.2441 (16) and 1.2488 (15) Å, indicate the delocalization of the negative charge.

The molecular components of the molecular salt are connected by two N^+^—H···O^-^ (±)CAHB hydrogen bonds to form ionic dimers with graph-set motif *R*^2^_2_(8) (Bernstein *et al.*, 1995). Furthermore, centrosymmetric ionic dimers are joined in ribbons running along the *b* axis by another *R*^2^_4_(8) motif involving the remaining N^+^—H···O^-^ hydrogen bonds (Fig. 2). Remarkably, at variance with the well known carboxylic dimer *R*^2^_2_ (8) motif, the carboxylate-amidinium pair is not planar, as the dihedral angle for the planes defined by the CN_2_^+^ and CO_2_^-^ atoms is equal to 18.57 (12)°.

### Experimental

Equimolar amounts (0.1 mmol) of benzamidine (Fluka at 96% purity) and 2-methoxybenzoic acid (Aldrich at 99% purity) were dissolved without further purification in 6 ml of hot water and heated under reflux for 6 h. After cooling the solution to an ambient temperature, colourless crystals suitable for single-crystal X-ray diffraction were grown by slow evaporation of the solvent after two weeks.

### Refinement

All H atoms were identified in difference Fourier maps, but for refinement all C-bound H atoms were placed in calculated positions, with C—H = 0.97 Å (phenyl) and 1.01 Å (methyl), and refined as riding on their carrier atoms. The *U*_iso_ values were kept equal to 1.2*U*_eq_(C, phenyl). and to 1.5*U*_eq_(C, methyl). The hydrogen atoms of the methyl group were allowed to rotate with a fixed angle around the C–C bond to best fit the experimental electron density [HFIX 138 in the *SHELX* program suite (Sheldrick, 2008)]. Positional and thermal parameters of H atoms of the amidinium group were freely refined, giving N—H distances in the range 0.86 (2) - 0.96 (2) Å.

### Crystal data

**Table:**

C_7_H_9_N_2_^+^·C_8_H_7_O_3_^−^	*Z* = 2
*M**_r_* = 272.30	*F*(000) = 288
Triclinic, *P*1	*D*_x_ = 1.267 Mg m^−^^3^
Hall symbol: -P 1	Mo *K*α radiation, λ = 0.71069 Å
*a* = 7.5154 (3) Å	Cell parameters from 9004 reflections
*b* = 9.1393 (4) Å	θ = 3.2–32.5°
*c* = 11.6498 (5) Å	µ = 0.09 mm^−^^1^
α = 69.612 (3)°	*T* = 298 K
β = 80.500 (5)°	Tablets, colourless
γ = 72.482 (4)°	0.12 × 0.09 × 0.05 mm
*V* = 713.63 (6) Å^3^	

### Data collection

**Table:**

Agilent Xcalibur Sapphire3 diffractometer	4332 independent reflections
Radiation source: Enhance (Mo) X-ray Source	3188 reflections with *I* > 2σ(*I*)
Graphite monochromator	*R*_int_ = 0.025
Detector resolution: 16.0696 pixels mm^-1^	θ_max_ = 30.5°, θ_min_ = 3.2°
ω and φ scans	*h* = −10→10
Absorption correction: multi-scan (*CrysAlis PRO*; Agilent, 2011)	*k* = −13→13
*T*_min_ = 0.989, *T*_max_ = 0.996	*l* = −16→16
20559 measured reflections	

### Refinement

**Table:**

Refinement on *F*^2^	Primary atom site location: structure-invariant direct methods
Least-squares matrix: full	Secondary atom site location: difference Fourier map
*R*[*F*^2^ > 2σ(*F*^2^)] = 0.062	Hydrogen site location: inferred from neighbouring sites
*wR*(*F*^2^) = 0.161	H atoms treated by a mixture of independent and constrained refinement
*S* = 1.05	*w* = 1/[σ^2^(*F*_o_^2^) + (0.0812*P*)^2^ + 0.1295*P*] where *P* = (*F*_o_^2^ + 2*F*_c_^2^)/3
4332 reflections	(Δ/σ)_max_ < 0.001
199 parameters	Δρ_max_ = 0.38 e Å^−^^3^
0 restraints	Δρ_min_ = −0.18 e Å^−^^3^

### Special details

**Table:**

Geometry. All s.u.'s (except the s.u. in the dihedral angle between two l.s. planes) are estimated using the full covariance matrix. The cell s.u.'s are taken into account individually in the estimation of s.u.'s in distances, angles and torsion angles; correlations between s.u.'s in cell parameters are only used when they are defined by crystal symmetry. An approximate (isotropic) treatment of cell s.u.'s is used for estimating s.u.'s involving l.s. planes.
Refinement. Refinement of *F*^2^ against ALL reflections. The weighted *R*-factor *wR* and goodness of fit *S* are based on *F*^2^, conventional *R*-factors *R* are based on *F*, with *F* set to zero for negative *F*^2^. The threshold expression of *F*^2^ > 2σ(*F*^2^) is used only for calculating *R*-factors(gt) *etc*. and is not relevant to the choice of reflections for refinement. *R*-factors based on *F*^2^ are statistically about twice as large as those based on *F*, and *R*- factors based on ALL data will be even larger.

### Fractional atomic coordinates and isotropic or equivalent isotropic displacement parameters (Å^2^)

**Table:**

	*x*	*y*	*z*	*U*_iso_*/*U*_eq_	
N1	−0.44074 (18)	0.33628 (15)	0.94036 (13)	0.0426 (3)	
H1A	−0.369 (3)	0.355 (2)	0.9866 (17)	0.052 (5)*	
H1B	−0.550 (3)	0.403 (2)	0.9196 (17)	0.053 (5)*	
N2	−0.21153 (17)	0.11282 (17)	0.93377 (13)	0.0464 (3)	
H2A	−0.139 (3)	0.138 (2)	0.9810 (17)	0.055 (5)*	
H2B	−0.167 (3)	0.022 (3)	0.9197 (18)	0.059 (5)*	
C1	−0.50196 (17)	0.16439 (15)	0.84181 (11)	0.0319 (3)	
C2	−0.6776 (2)	0.2674 (2)	0.81080 (18)	0.0531 (4)	
H2	−0.7178	0.3693	0.8281	0.064*	
C3	−0.7954 (2)	0.2254 (2)	0.75542 (18)	0.0602 (5)	
H3	−0.9172	0.2986	0.7333	0.072*	
C4	−0.7414 (2)	0.0816 (2)	0.73164 (15)	0.0508 (4)	
H4	−0.8254	0.0522	0.6939	0.061*	
C5	−0.5678 (2)	−0.0216 (2)	0.76121 (16)	0.0504 (4)	
H5	−0.5291	−0.1233	0.7435	0.060*	
C6	−0.4480 (2)	0.01930 (18)	0.81621 (14)	0.0418 (3)	
H6	−0.3257	−0.0539	0.8369	0.050*	
C7	−0.38026 (17)	0.20627 (15)	0.90698 (12)	0.0323 (3)	
O1	−0.24816 (15)	0.39582 (13)	1.09618 (11)	0.0513 (3)	
O2	−0.00870 (15)	0.18920 (14)	1.08560 (11)	0.0536 (3)	
O3	−0.24579 (19)	0.2238 (2)	1.37136 (12)	0.0694 (4)	
C8	−0.00944 (18)	0.31556 (15)	1.23202 (12)	0.0335 (3)	
C9	−0.0897 (2)	0.2793 (2)	1.35187 (14)	0.0434 (3)	
C10	−0.0097 (3)	0.2988 (2)	1.44339 (15)	0.0572 (4)	
H10	−0.0654	0.2737	1.5268	0.069*	
C11	0.1493 (3)	0.3540 (2)	1.41503 (17)	0.0578 (5)	
H11	0.2044	0.3678	1.4790	0.069*	
C12	0.2300 (3)	0.3895 (2)	1.29805 (18)	0.0567 (4)	
H12	0.3417	0.4281	1.2788	0.068*	
C13	0.1494 (2)	0.3694 (2)	1.20632 (14)	0.0466 (4)	
H13	0.2066	0.3940	1.1233	0.056*	
C14	−0.09582 (18)	0.29772 (15)	1.13074 (12)	0.0335 (3)	
C15	−0.3237 (4)	0.1697 (4)	1.4934 (2)	0.0938 (8)	
H15A	−0.2241 (19)	0.083 (2)	1.5466 (11)	0.141*	
H15B	−0.430 (3)	0.123 (2)	1.4938 (3)	0.141*	
H15C	−0.372 (3)	0.2639 (17)	1.5268 (9)	0.141*	

### Atomic displacement parameters (Å^2^)

**Table:**

	*U*^11^	*U*^22^	*U*^33^	*U*^12^	*U*^13^	*U*^23^
N1	0.0387 (6)	0.0361 (6)	0.0566 (8)	0.0057 (5)	−0.0185 (5)	−0.0255 (6)
N2	0.0370 (6)	0.0443 (7)	0.0641 (8)	0.0087 (5)	−0.0192 (6)	−0.0341 (7)
C1	0.0328 (6)	0.0316 (6)	0.0304 (6)	−0.0023 (5)	−0.0065 (5)	−0.0122 (5)
C2	0.0485 (8)	0.0434 (8)	0.0714 (11)	0.0097 (7)	−0.0279 (8)	−0.0309 (8)
C3	0.0469 (9)	0.0595 (10)	0.0779 (12)	0.0077 (8)	−0.0332 (8)	−0.0317 (9)
C4	0.0538 (9)	0.0555 (9)	0.0506 (9)	−0.0146 (7)	−0.0188 (7)	−0.0189 (7)
C5	0.0590 (9)	0.0429 (8)	0.0573 (9)	−0.0076 (7)	−0.0158 (7)	−0.0253 (7)
C6	0.0410 (7)	0.0373 (7)	0.0483 (8)	0.0006 (6)	−0.0120 (6)	−0.0202 (6)
C7	0.0325 (6)	0.0305 (6)	0.0331 (6)	−0.0015 (5)	−0.0057 (5)	−0.0133 (5)
O1	0.0498 (6)	0.0453 (6)	0.0616 (7)	0.0158 (5)	−0.0293 (5)	−0.0328 (5)
O2	0.0462 (6)	0.0537 (7)	0.0690 (7)	0.0137 (5)	−0.0211 (5)	−0.0442 (6)
O3	0.0628 (8)	0.1107 (12)	0.0494 (7)	−0.0439 (8)	0.0064 (6)	−0.0300 (7)
C8	0.0341 (6)	0.0297 (6)	0.0363 (6)	0.0028 (5)	−0.0105 (5)	−0.0156 (5)
C9	0.0415 (7)	0.0506 (8)	0.0409 (7)	−0.0066 (6)	−0.0066 (6)	−0.0208 (6)
C10	0.0624 (10)	0.0752 (12)	0.0384 (8)	−0.0115 (9)	−0.0104 (7)	−0.0258 (8)
C11	0.0645 (11)	0.0638 (11)	0.0548 (10)	−0.0087 (9)	−0.0253 (8)	−0.0279 (8)
C12	0.0536 (9)	0.0614 (10)	0.0652 (11)	−0.0192 (8)	−0.0166 (8)	−0.0232 (9)
C13	0.0472 (8)	0.0541 (9)	0.0420 (8)	−0.0150 (7)	−0.0045 (6)	−0.0175 (7)
C14	0.0344 (6)	0.0301 (6)	0.0366 (6)	−0.0001 (5)	−0.0088 (5)	−0.0156 (5)
C15	0.0826 (16)	0.144 (2)	0.0608 (13)	−0.0546 (17)	0.0201 (12)	−0.0299 (14)

### Geometric parameters (Å, º)

**Table:**

N1—C7	1.3070 (17)	O1—C14	1.2488 (15)
N1—H1A	0.913 (19)	O2—C14	1.2441 (16)
N1—H1B	0.87 (2)	O3—C9	1.369 (2)
N2—C7	1.3145 (16)	O3—C15	1.421 (3)
N2—H2A	0.959 (19)	C8—C13	1.375 (2)
N2—H2B	0.86 (2)	C8—C9	1.394 (2)
C1—C6	1.3869 (19)	C8—C14	1.5135 (17)
C1—C2	1.3898 (19)	C9—C10	1.393 (2)
C1—C7	1.4838 (18)	C10—C11	1.381 (3)
C2—C3	1.383 (2)	C10—H10	0.9700
C2—H2	0.9700	C11—C12	1.367 (3)
C3—C4	1.366 (2)	C11—H11	0.9700
C3—H3	0.9700	C12—C13	1.399 (2)
C4—C5	1.375 (2)	C12—H12	0.9700
C4—H4	0.9700	C13—H13	0.9700
C5—C6	1.386 (2)	C15—H15A	1.0120
C5—H5	0.9700	C15—H15B	1.0120
C6—H6	0.9700	C15—H15C	1.0120
			
C7—N1—H1A	119.2 (12)	C13—C8—C9	119.07 (13)
C7—N1—H1B	120.3 (12)	C13—C8—C14	120.07 (12)
H1A—N1—H1B	120.5 (17)	C9—C8—C14	120.86 (12)
C7—N2—H2A	119.6 (11)	O3—C9—C10	124.03 (15)
C7—N2—H2B	123.2 (13)	O3—C9—C8	115.99 (13)
H2A—N2—H2B	116.7 (17)	C10—C9—C8	119.97 (15)
C6—C1—C2	118.65 (13)	C11—C10—C9	119.83 (16)
C6—C1—C7	121.19 (11)	C11—C10—H10	120.1
C2—C1—C7	120.08 (12)	C9—C10—H10	120.1
C3—C2—C1	120.39 (14)	C12—C11—C10	120.83 (15)
C3—C2—H2	119.8	C12—C11—H11	119.6
C1—C2—H2	119.8	C10—C11—H11	119.6
C4—C3—C2	120.44 (15)	C11—C12—C13	119.22 (16)
C4—C3—H3	119.8	C11—C12—H12	120.4
C2—C3—H3	119.8	C13—C12—H12	120.4
C3—C4—C5	119.99 (14)	C8—C13—C12	121.07 (15)
C3—C4—H4	120.0	C8—C13—H13	119.5
C5—C4—H4	120.0	C12—C13—H13	119.5
C4—C5—C6	120.16 (15)	O2—C14—O1	124.40 (12)
C4—C5—H5	119.9	O2—C14—C8	118.04 (11)
C6—C5—H5	119.9	O1—C14—C8	117.53 (11)
C5—C6—C1	120.36 (13)	O3—C15—H15A	109.5
C5—C6—H6	119.8	O3—C15—H15B	109.5
C1—C6—H6	119.8	H15A—C15—H15B	109.5
N1—C7—N2	118.81 (13)	O3—C15—H15C	109.5
N1—C7—C1	120.25 (11)	H15A—C15—H15C	109.5
N2—C7—C1	120.93 (12)	H15B—C15—H15C	109.5
C9—O3—C15	118.79 (15)		
			
C6—C1—C2—C3	−0.1 (3)	C14—C8—C9—O3	1.6 (2)
C7—C1—C2—C3	−176.85 (16)	C13—C8—C9—C10	0.4 (2)
C1—C2—C3—C4	0.7 (3)	C14—C8—C9—C10	−179.03 (14)
C2—C3—C4—C5	−0.9 (3)	O3—C9—C10—C11	179.31 (17)
C3—C4—C5—C6	0.6 (3)	C8—C9—C10—C11	−0.1 (3)
C4—C5—C6—C1	0.0 (3)	C9—C10—C11—C12	−0.2 (3)
C2—C1—C6—C5	−0.2 (2)	C10—C11—C12—C13	0.1 (3)
C7—C1—C6—C5	176.50 (14)	C9—C8—C13—C12	−0.6 (2)
C6—C1—C7—N1	−173.63 (14)	C14—C8—C13—C12	178.91 (14)
C2—C1—C7—N1	3.1 (2)	C11—C12—C13—C8	0.3 (3)
C6—C1—C7—N2	5.4 (2)	C13—C8—C14—O2	68.85 (18)
C2—C1—C7—N2	−177.93 (15)	C9—C8—C14—O2	−111.69 (16)
C15—O3—C9—C10	−5.5 (3)	C13—C8—C14—O1	−109.51 (16)
C15—O3—C9—C8	173.93 (19)	C9—C8—C14—O1	69.95 (18)
C13—C8—C9—O3	−178.98 (14)		

### Hydrogen-bond geometry (Å, º)

**Table:**

*D*—H···*A*	*D*—H	H···*A*	*D*···*A*	*D*—H···*A*
N1—H1*A*···O1	0.913 (19)	1.87 (2)	2.7777 (16)	172.4 (17)
N1—H1*B*···O1^i^	0.87 (2)	1.97 (2)	2.7926 (15)	155.7 (17)
N2—H2*A*···O2	0.959 (19)	1.93 (2)	2.8863 (17)	175.4 (16)
N2—H2*B*···O2^ii^	0.86 (2)	2.00 (2)	2.8230 (16)	160.3 (18)

Symmetry codes: (i) −*x*−1, −*y*+1, −*z*+2; (ii) −*x*, −*y*, −*z*+2.
